# Flexible Thin Film Functionalized by Initiative Dust Removal and Anti-Fogging for Optical Device Applications

**DOI:** 10.3390/s24010057

**Published:** 2023-12-21

**Authors:** Yingqi Feng, Li Tian, Zunkai Huang, Chenghe Yang, Linhai Guo, Yuwei Jiang, Chenye Wei, Yu Guo, Hui Wang

**Affiliations:** 1 Shanghai Advanced Research Institute, Chinese Academy of Sciences, Shanghai 201210, China; fengyq@sari.ac.cn (Y.F.); wanghui@sari.ac.cn (H.W.); 2 University of Chinese Academy of Sciences, Beijing 100049, China; 3 Shanghai Nuclear Engineering Research & Design Institute Co., Ltd., Shanghai 200233, China

**Keywords:** electrodynamic screen (EDS), signal-to-noise ratio (PSNR), film-driven algorithm

## Abstract

The deposition of dust and condensation of fog will block the scattering and transmission of light, thus affecting the performance of optical devices. In this work, flexible polyethylene terephthalate (PET) foil functionalized by active dust removal and anti-fogging characteristics is realized which combines electrodynamic screen (EDS) and electro-heating devices. In lieu of traditional measurement methods of dust removal efficiency, the PSNR is employed to characterize the dust removal efficiency of the film for the first time. The results show that both dust removal and anti-fogging improve the image quality, in which the dust removal increases the PSNR from 28.1 dB to 34.2 dB and the anti-fogging function realizes a film temperature rise of 16.7 ${}^{\circ }$C in 5 min, reaching a maximum of 41.3 ${}^{\circ }$C. According to the high sensitivity of the PSNR, we propose a fully automatic CIS film-driven algorithm, and its feasibility has been demonstrated.

## 1. Introduction

In recent years, functional optical devices have become a research hotspot due to their widespread applications in various fields such as artificial intelligence, neuromorphic computing, signal processing, optical sensing, and optical communication systems [1,2,3,4,5,6,7]. These devices are generally covered by optical glass to protect them against external shocks and moisture. The glass surface will inevitably be affected by dust accumulation or water vapor condensation (fogging), which will block or scatter incoming light [8,9]. This phenomenon is prevalent and could lead to performance degradation, which is also one of the concerns in the photovoltaic community [10,11]. For example, when dust deposits on the cover glass of an infrared camera, the measurement accuracy of the infrared camera will be disturbed due to the temperature drop of the black body [12]. As dust accumulates on the cover glass of the photovoltaic module, it forms a thin layer that reduces the transmittance of the glass by reflecting, absorbing, and scattering light, thus affecting the efficiency and thus the power output [10,13]. In addition, in the medical field, the presence of condensation on an endoscope lens will result in a decline in image quality so that a clear view cannot be provided, which is an important obstacle to the safety and success of the operation [8,14]. In almost all optical applications which require materials having excellent optical properties, the optical surface should be kept clean to prevent dust deposition or the occurrence of the fogging phenomenon.

A series of active and passive cleaning and anti-fog methods have been proposed to address the above issues. Active dust cleaning methods, including wiping, blowing, and ultrasonic driving, achieve the goal of dust removal [15,16,17,18,19], but there are problems such as low dust removal efficiency, poor economy, and the potential for surface damage to devices. Active anti-fogging technology, which involves regulating air velocity and surface temperatures, effectively prevents fogging. However, its implementation commonly leads to heightened energy consumption and maintenance expenses [20,21]. The passive schemes involve regulating surfaces with (super)hydrophilic and (super)hydrophobic characteristics via micro(nano)-structures or functional coatings [16,22,23,24,25], where the hydrophilicity serves to form a uniform water film on the surface and the hydrophobicity serves to increase the contact angle of water droplets [10,26,27,28]. Xiaolei Luo et al. demonstrate biomimetic moth-eye structures on glass using an inductively coupled plasma (ICP) etching process, which possesses superhydrophilic self-cleaning and anti-fogging abilities [23]. Researchers manufacture a superhydrophobic surface with nanostructures containing fluorinated polymers, exhibiting a contact angle (CA) with water droplets ranging from 149${}^{\circ }$ to 151${}^{\circ }$ [28]. However, all the active dust self-cleaning strategies mentioned above will encounter mechanical instability and poor economy issues. For passive strategies, the above-mentioned micro(nano)-structures or functional coatings are easily damaged in the harsh outdoor environment, thereby reducing their service life. In particular, the micro(nano)-structures need to be in the order of microns, which will scatter light and is not suitable for optical sensors.

Alternatively, as an active cleaning technology, electrodynamic screen (EDS) technology has been proposed on the photovoltaic modules in both terrestrial and space applications [15,29,30,31,32,33,34]. The dust particles accumulated on the film surface are charged and electrostatically repelled by the multi-phase high-voltage pulses realized by a set of parallel electrodes. This method relies on electrostatic force to achieve the purpose of water-free and mechanical motion-free dust removal. Up to 98% dust removal efficiency was achieved by the EDS technology [35], which also offers maintenance-free features due to the absence of mechanical movements. However, such a method will be affected by relative humidity [33]. When relative humidity exceeds a certain threshold, the capillary force is one order of magnitude higher than the van der Waals force, so the Coulomb force is ineffective in removing fine dust particles. Furthermore, the optical transparency of the electrode is also a point that needs to be paid attention to [36].

In this work, flexible polyethylene terephthalate (PET) foil functionalized by active dust removal and anti-fogging characteristics is realized on devices; it combines EDS and electro-heating. The two devices are realized on each side of the PET foils by pattering indium tin oxide (ITO) coating thin film, which is a well-known transparent conductive material. The spiral pattern with a three-phase concentric electrode with a width no more than 100 μm is realized by the laser scribing method. The anti-fogging function achieved by full-area ITO coating prevents the water condensation and helps improve the dust removal efficiency at the same time. The electronic driving system is realized by a controller, a power management module, and MOSFET relays. With the assistance of a captured image, the peak signal-to-noise ratio (PSNR) is employed for the first time to evaluate the dust removal efficiency (DRE), in lieu of the conventionally used gravimetric and photometric methods [30,37,38]. As a demonstration, the dual-functional films are systematically evaluated on a CMOS image sensor, which can be facially attached onto the cover glass. In addition, the change in PSNR can be used as a quantitative parameter to determine when the dust removal function or anti-fogging function is triggered, which is attractive for outdoor applications.

## 2. Materials and Methods

### 2.1. Methods and Simulation

**Figure 1:**
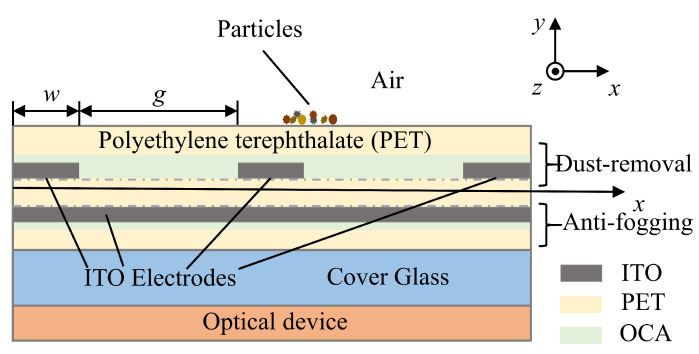
A cross-schematic illustration of flexible thin film functionalized with active dust removal and anti-fogging modules.

The cross-section of a flexible PET foil functionalized with active dust removal and anti-fogging modules is illustrated in Figure 1, where three parallel spiral electrodes are functionalized as the dust removal module on the front side, and the anti-fogging module is composed of a full-area ITO coating on the rear side. The width of the spiral electrodes and inter-electrode spacing are, respectively, denoted as w and g. The upper side of the spiral electrodes is covered by a blank PET foil bonded with an optically clear adhesive (OCA) film, which can protect the ITO electrodes and provide a high dielectric constant to avoid air breakdown [39]. The free-standing flexible film can work independently or can be attached to the glass window of an optoelectronic device.

The removal of surface particles is a process of competition between adhesion and repulsion forces. When the electric field at the edge of the electrode increases to the initial point of surface breakdown, particles that accumulate on the surface of the optical element covered by the electrode will become positively charged, due to the formation of a weak ionizing field [33,39,40]. The adhesion ($F_{adh}$) and repulsion ($F_{req}$) forces exerted on surface particles can be calculated as follows:

(1) $$F_{adh}=\left\{\begin{array}{cc} F_{vdw}+F_{im}+F_{g}+F_{cap}, & if\;RH\;>\;40\%\\ F_{vdw}+F_{im}+F_{g}, & else \end{array}\right.$$

(2) $$F_{rep}=F_{C}.$$

where contributions by van der Waals force ($F_{vdw}$), image force ($F_{im}$), gravitational force ($F_{g}$), and capillary force ( $F_{cap}$) are considered. $F_{cap}$ can be calculated from Equation (3); the equilibrium radius of the meniscus *r* can be calculated by the equation $r=-(\gamma_{W}\;V_{water})/$$\left(N_{a}kTlnRH\right)$, in which $\gamma_{W}$ and $V_{water}$ represent the surface tension of water at room temperature ($\gamma_{W}=0.073$ N/m) and the molar volume of water ($V_{water}=18\times 10^{-6}$ m${}^{3}$/mol). Apart from this, θ represents the contact angles of particle and surface. It is assumed that the $F_{cap}$ is negligible when the relative humidity (RH) is less than 40% [33].

(3) $$F_{cap}=2\pi \gamma_{W}dcos\theta \left[1-(\frac{R_{a}}{rcos\theta })\right].$$

When the repulsion force $F_{C}$ is greater than the sum of $F_{adh}$, the dust particles will leave the surface vertically. It should be noted that when neutral particles are in a non-uniform electric field, the induced dipole moment generated inside the particles interacts with the electric field to form a dielectrophoretic (DEP) force [41], which makes the particles move to the stronger or weaker electric field. Under the combined effects of the DEP force and Coulomb force, a traveling wave will be generated in the horizontal direction. Then, dust particles will be removed from the surface of the electrode to achieve the purpose of dust removal. When the electric field is computed in the frequency domain, the DEP force adds the following contribution to the total forces exerted on the particles:

(4) $$F_{d}=\left(P\cdot \nabla \right)E=\frac{1}{4}\pi d^{3}\varepsilon_{0}real\left(\varepsilon_{r}^{*}\right)real\left(\frac{\varepsilon_{r,p}^{*}-\varepsilon_{r}^{*}}{\varepsilon_{r,p}^{*}+2\varepsilon_{r}^{*}}\right)\nabla {\left|E_{rms}\right|}^{2}$$

in which *P* is the effective dipole moment and will disappear when the external electric field is removed. The $E_{rms}$ is the root mean square electric field.

**Figure 2:**
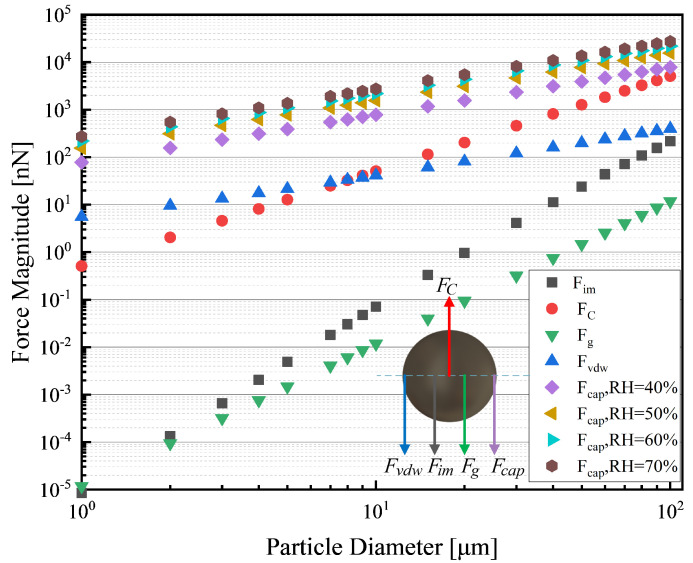
Comparison of the adhesion and repulsion forces exerted on the particle and capillary force for several different RH values.

The adhesion ($F_{adh}$) and repulsion forces ($F_{C}$) as functions of particle diameters are shown in Figure 2, where the $F_{cap}$ is calculated at the RH of 40%, 50%, 60%, and 70%. It is easy to see that $F_{C}$ is greater than the sum of the various $F_{adh}$ without the capillary force and causes the dust particles to leave the optical surface vertically. However, if the RH of the surface is high (RH > 40%), the $F_{adh}$ will grow so rapidly that the process of dust removal is inhibited. Furthermore, it is recognized that fog can adversely affect the optical performance of the device and enhance the $F_{cap}$ exerted on the particles, which will hinder the process of dust removal [36].

In order to solve the above problems, this work utilizes the electrothermal efficiency of the transparent metal film to reduce the negative effects of fog. Specifically, a voltage is applied to the ITO thin film resistive layer, which generates Joule heat to achieve the purpose of removing water mist and reducing relative humidity. If voltage $V_{low}$ is applied on it, the rate of heat consumption per unit area $q_{prod}$ (W/m${}^{2}$) is given by:

(5) $$q_{prod}=dQ_{DC}$$

in which power density $Q_{DC}$ can be calculated by the equation $Q_{DC}=J\cdot \;E=\sigma \;{\left|\nabla_{t}\cdot \;V_{low}\;\right|}^{2}$, where σ is the conductivity of ITO.

**Figure 3:**
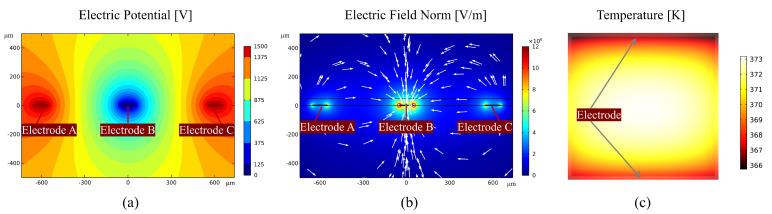
Numerical simulation results using finite element analysis software. (**a**) Electric potential in one fundamental spatial period for the dust removal model. (**b**) Electric field norm in one fundamental spatial period for the dust removal model. (**c**) Temperature in the steady state for the anti-fogging module.

While $F_{C}$ is the guarantee of EDS technology, the dielectrophoretic force ($F_{d}$) is the key factor for dust removal. Both the direction and magnitude of the force $F_{d}$ depend upon the electric field magnitude. In order to corroborate the spatial electric field distribution, the active dust removal module with one layer of PET has been developed in COMSOL Multiphysics^®^. The w, g, and thickness of the dielectric coating (PET) are 100 μm, 500 μm, and 40 μm, respectively. The relative permittivity of the PET coating is 3. The electric potential *U* and the electric field magnitude $\left|E\right|={\left(E_{x}^{2}+E_{y}^{2}\right)}^{0.5}\;$ on the surface in one fundamental spatial period are in Figure 3a,b. The *U* of either electrodes A or C is 1.5 kV, which is the upper voltage limit of the electronic driving system mentioned in Section 2.3, while the *U* of electrode B is set to 0 V. It can be inferred that the electric field vectors in Figure 3b originate from electrodes A and C and terminate in electrode B. The |E| reaches its maximum value of 4.929 MV/m on the EDS surface just above the surface of electrode B in one fundamental spatial period.

Similarly, the anti-fogging module composed of a layer of ITO coating with a square resistance of 40 Ω/sq has been simulated in COMSOL Multiphysics^®^. The size of the module is 4 cm × 4 cm, and the positive and negative electrodes are, respectively, located above and below the module. The distribution of temperature is shown in Figure 3c. In the steady state, the maximum temperature can reach 373 K in the center of the module, which can prevent the process of water vapor condensation effectively.

### 2.2. New Efficiency Evaluation Method

**Figure 4:**
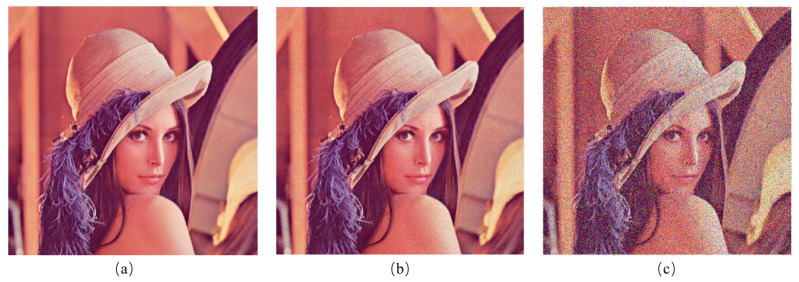
Simulation of dust particles by salt and pepper noise and Poisson noise on an image. (**a**) Raw image without any processing. (**b**) Image with Poisson noise. (**c**) Image with Poisson noise, salt and pepper noise (SNR = 0.6).

Traditional efficiency evaluation methods such as the gravimetric, surface area, and photometric methods are complex for the evaluation of dust removal efficiency [30,37,42]. The peak signal-to-noise ratio (PSNR) is applied in this work as an efficiency evaluation method to measure the function of dust removal, as the removal process can be seen as a noise reduction process. PSNR is based on the error between pixels [43,44], namely based on error-sensitive image quality assessment, which is more sensitive to dust particles compared to traditional methods. Suppose that the dust particles on the image are a combination of salt and pepper noise (Signal Noise Rate, SNR = 0.6) and Poisson noise (Figure 4), caused by the statistical nature of light and the photoelectric conversion process of image sensors. The probability functions of salt and pepper noise and Poisson noise are calculated as follows:

(6) $$P_{z}=\left\{\begin{array}{cc} P_{a}, & z=white(salt)\;\\ P_{b}, & z=black(pepper)\;\\ 1-P_{a}-P_{b}=1-\mathrm{SNR}, & else \end{array}\right.$$

(7) $$P\left(X=k\right)=\frac{e^{-\lambda }\lambda^{k}}{k!}$$

where z represents random locations where noise occurs, and the total number of *a* and *b* is indicated by the SNR. In Equation (7), *k* stands for event and λ represents the average probability of random events per unit area. In order to apply PSNR to evaluation, we collect the images with pixel size of m×n before and after the system works as the image I and K. Firstly, the mean square error (MSE) between the reference image and the noise image is calculated using the following equation:

(8) $$\mathrm{MSE}=1/mn\;\sum_{i=0}^{m-1}\;\sum_{j=0}^{n-1}{\left[I\left(i,j\right)-K\left(i,j\right)\right]}^{2}$$

where i and j are the abscissa and ordinate of the pixel in the image. Secondly, the PSNR according to the MSE value is counted in dB:

(9) $$\mathrm{PSNR}=10\times \;log_{10}{\left(2^{B}-1\right)}^{2}/\mathrm{MSE}$$

where *B* is the number of bits for every sampling value, generally taking 8. According to the above formula, it can be inferred that the larger the PSNR value, the better the effect of the flexible film in removing dust.

### 2.3. Design of Electronic Driving System

**Figure 5:**
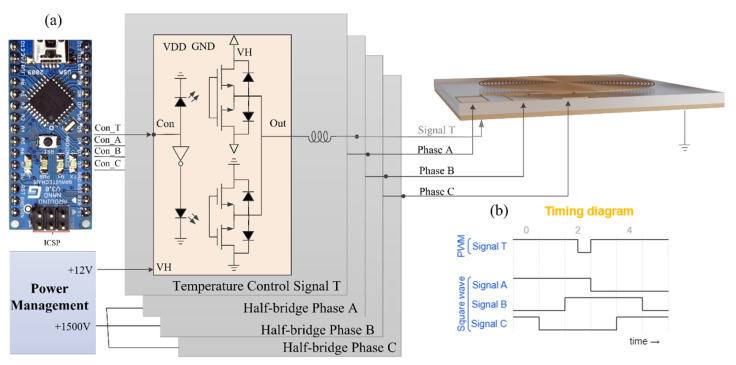
Schematic illustrations of functionalized thin film system design. (**a**) Electronic driving system design. (**b**) Timing diagram for activation of functionalized thin film.

The customized electronics system for driving the functionalized film is shown in Figure 5a, consisting of a controller, power management module, and MOSFET relays. The system is highly integrated and easy to carry and operate. Best of all, the system requires no external large-scale power supply. Specifically, the controller uses the open-source electronic prototyping platform Arduino. Its cross-platform and open-source features build the foundation for the miniaturization of the system. The power management module provides a stable voltage of 5 V and a high voltage of 1500 V (1 mA), in which a cascaded boost converter is used to achieve stable conversion from low voltage to high voltage. It is worth noting that this design replaces the reed relays by combining MOSFETs as relays, which are superior to reed relays in terms of switching speed and withstanding voltage.

From the circuit point of view, the relays used for both the dust removal module and anti-fogging module are the same. The schematic illustration of the MOSFET relays is shown in Figure 5a. MOSFET relays use extremely low (almost zero) electrical energy to control the switching of both low and high voltages. For the electrothermal anti-fogging module of the functionalized film, the controller outputs the signal T, as shown in Figure 5b. Pulse width modulation (PWM) is performed by controlling the on–off time of the MOSFET relay to achieve the purpose of controlling the temperature. For the dust removal module, the controller outputs three-phase square waves, as shown in Figure 5b. The three-phase high-voltage signal is generated through the combination of MOSFET relays to achieve the effect of removing dust particles. The frequency of the square waves corresponds to the operating frequency of the system, generally set at 5–20 Hz [32,42,45].

### 2.4. Film Design

**Figure 6:**
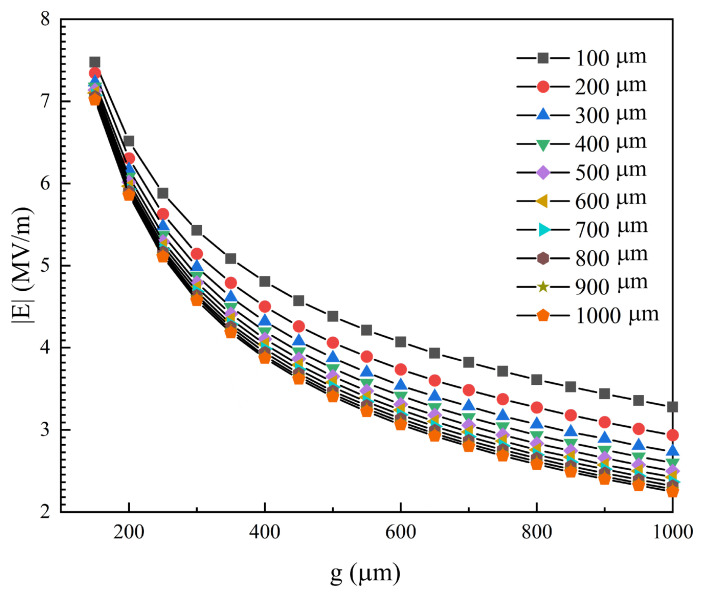
The impact of the electrode parameters on the maximum of electric field norm.

Exploring the design method of electrode parameters is of great significance for improving the dust removal efficiency of EDS. As is derived in the Experimental Section, we use electric field magnitude to evaluate the influence of electrode parameters in the dust removal module. The impacts of the electrode parameters (w, g) on the maximum of the electric field magnitude |E| on the surface in one fundamental spatial period are shown in Figure 6. As the inter-electrode spacing g decreases, the maximum of |E| increases obviously. For instance, when w = 500 μm and g change from 150 μm to 1000 μm, the electric field magnitude increment is a multiple of 2.85. As highlighted in Figure 6, when either w or g decreases, |E| will increase so that the function of dust removal is easier to activate. However, experiments show that there are lower limits for both w and g, and the dust cannot escape the shackles of the electric field under the conditions of small electrode parameters (w, g) [46]. Comprehensively considering the effects of the electrode parameters (w, g) on the function and stability, the design parameters are set as w = 100 μm and g = 500 μm in this work.

### 2.5. Film-Driven Algorithm Design

**Figure 7:**
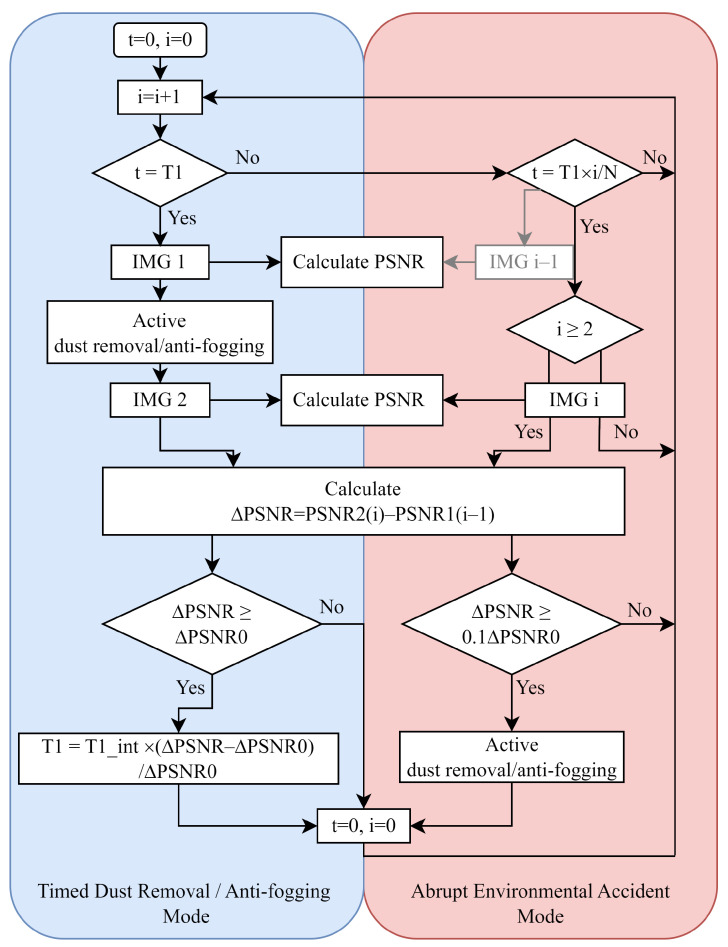
The flow of fully automatic CIS dust removal/anti-fogging algorithm based on PSNR.

In addition to the evaluation of dust or fog, PSNR can also be used to guide the functional triggering of the functional thin films manufactured in this work. This research provides a promising fully automatic CIS film-driven algorithm based on PSNR, which can drive single or multiple functions and is highly scalable. The fully automatic CIS dust removal/anti-fogging algorithm is shown in Figure 7. The algorithm takes the impact of environmental factors into account and can make corresponding judgments for situations with low, high, and rapid dust deposition. Taking the dust removal module as a demonstration, the algorithm consists of two modes: Adaptive Timing Mode and Abrupt Environmental Accident (AEA) Mode. When the film system is activated, both modes operate in parallel.

In the context of the adaptive timing mode, at regular intervals of time T1, the system drives the CIS to take a frame of image IMG1 and calculate its PSNR (PSNR1). At the same time, the function of dust removal is activated. After dust removal is completed, the system drives the CIS to obtain a frame of photo IMG2 and calculates its PSNR(PSNR2). The PSNR difference value of these two frames of photos is calculated as ΔPSNR=PSNR2−PSNR1. It is assumed that the CIS shooting content does not change before or after dust removal, and the CIS shooting effect returns to a dust-free level after dust removal. From this perspective, ΔPSNR not only represents the dust removal effect but also represents the amount of dust Qdust on the CIS during time T1. It is worth mentioning that the algorithm can optimize the value of T1 based on the value of ΔPSNR. When a large amount of dust causes ΔPSNR to exceed ΔPSNR0 in T1, the value of T1 will decrease to boost the frequency of scheduled dust removal operations. T1 is calculated as:

(10) $$T1=T1_{int}*\left(\Delta PSNR-\Delta PSNR0\right)/\Delta PSNR0$$

where $T1_{int}$ is the system’s preset timing time and ΔPSNR0 is the reference to adjust T1. ΔPSNR represents the PSNR differences calculated successively, which is continuously updated as the system operates.

Correspondingly, when an abrupt environmental accident occurs, such as a sudden increase in dust deposition caused by a sandstorm or passing vehicle, the proposed AEA Mode will activate. The condition for for starting is the detection of an increase in dust in a short period of time through the CIS. Specifically, a frame of photo IMG i is obtained after a short period of time (1/N)∗T1 and the PSNR value (PSNRi) will be calculated. *N* represents the times that AEA Mode runs during T1. The difference between PSNRi and PSNR(i−1) obtained in the previous period is ΔPSNR. If ΔPSNR exceeds 0.1∗ΔPSNR0, which means a large amount of dust deposition within a short time of (1/N)∗T1, the dust removal operation will be triggered and the timer *t* will be reset.

## 3. Results and Discussion

As a representative of transparent metal oxides, ITO has good optical and electrical properties. To explore the optical properties of ITO-based functionalized flexible film, we test the transmittance of normal silica-based glass and the proposed flexible thin film using the Solar Cell Spectral Response Measurement System Model QEX10 (PV measurements, Boulder, CO, USA). On the other hand, we discuss the performance of thin film electrical aspects. The dust removal module of the film is verified by the CMOS Image Sensor (CIS) using 0.18 μm CIS technology. To verify whether PSNR is reasonable as a measure of dust removal efficiency, we take photos under different dust deposition conditions and water condensation through CIS. The anti-fogging module of the film proposed in this paper is verified by UTi260B (UNI-T Company, Dongguan, China).

**Figure 8:**
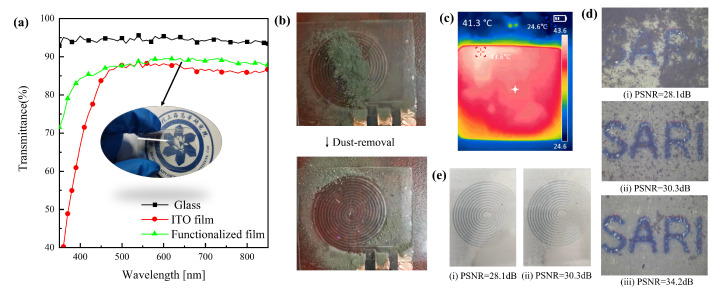
Experimental results of functionalized thin films and the illustration of PSNR for dust removal efficiency. (**a**) The comparison of transmittance on quartz glass, uniform layer of ITO, ITO thin film patterned by laser etching. (**b**) Demonstration of the proposed film dust removal module. (**c**) Demonstration of the proposed film electric heating anti-fogging module. (**d**) The comparison of PSNR under different dust deposition conditions. (**e**) The comparison of PSNR under different fogging phenomenon conditions.

For optical device applications such as camera lenses, it is necessary for thin film to have a high transmittance. Figure 8a shows the comparison of transmittance on quartz glass, uniform ITO film, and the functionalized PET film. The film exhibits excellent optical performance, with a transmission rate of nearly 90% in the wavelength range of 400 to 850 nm, making it suitable for use in optical devices.

According to the above simulation results, the electronic driving system mentioned above is used to verify the characteristics of film dust removal and anti-fogging, respectively. Particles which originate from the air and the earth’s surface are mainly composed of quartz (SiO_2_), calcite (CaCO_3_), and orthoclase (NaAlSi_3_O_8_) [47,48]. In order to facilitate the construction of three parallel electrodes in a plane, the electrode shape is designed as a spiral structure. Figure 8b demonstrates the dust removal performance of the film. It can be seen that dust is transported to the edge of the film quickly under the driving of three-phase high-voltage (1.5 KV, 10 Hz) pulses. Similarly, the electronic driving system is used to activate the film function of anti-fogging. As shown in Figure 8c, the center temperature of the film reaches 41.3 ${}^{\circ }$C after 5 min of activation with the PWM (duty cycle set to 70%) wave. In this case, the process of water condensation will be disrupted, which avoids the adverse effects of fog.

**Figure 9:**
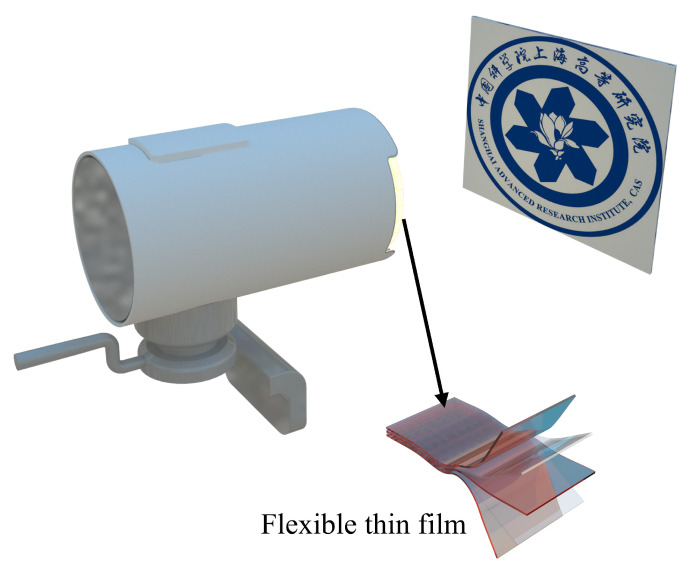
Schematic illustration of the application of functionalized flexible films to optical devices.

The proposed index PSNR can be used in optical systems to guide functionalized film work effectively. As a demonstration, the film is attached onto the cover glass of CIS. We build the experimental system as shown in Figure 9, which is made up of a pattern plate, flexible film, and CIS (CIS is packaged with the cover glass).

**Figure 10:**
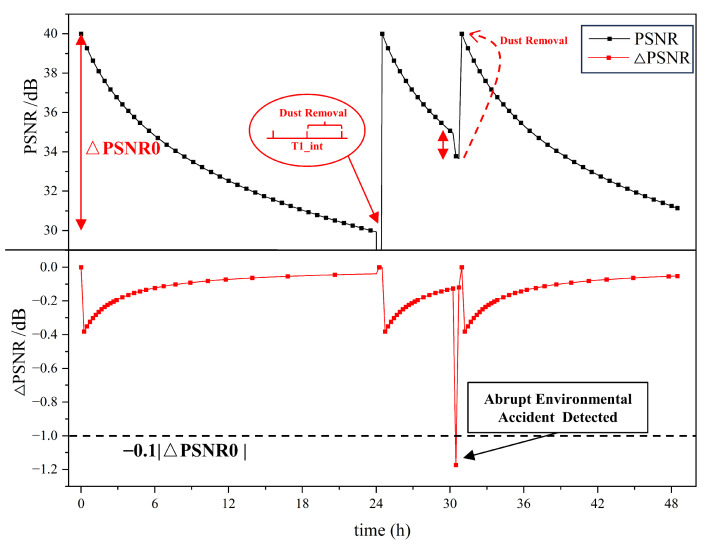
The process of guidance according to the PSNR.

The results of the automatic CIS dust removal algorithm are shown in Figure 10. The initial timing length $T1_{int}$ is set to 24 h. The times of detection (*N*) of AEA within the period is 100, and the reference PSNR difference (ΔPSNR0) of the period is set to 10 dB. The system is in an environment where the amount of dust is deposited linearly. According to Equation (9), the change in PSNR is logarithmic. It shows that no abrupt environmental accident was detected in the previous 24 h, and the timed dust removal routine was successfully activated when $=T1_{int}$. After this, the PSNR returns to the dust-free level and starts timing and depositing dust again. An accident was detected at *t* = 30.2 h, which activated the dust removal module and pulled the PSNR back to the dust-free level again.

## 4. Conclusions

For optical device application, this paper introduces a functionalized flexible film with initiative dust removal and anti-fogging characteristics. Both the function of dust-removal and the function of anti-fogging are activated by the self-designed electronic driving system. In lieu of traditional measurement methods of dust removal efficiency, the PSNR is employed to characterize the dust removal efficiency of the film for the first time, which has the advantages of high sensitivity and wide application. The experimental results show that both dust removal and anti-fogging functions improve the image quality, in which the dust removal increases the PSNR from 28.1 dB to 34.2 dB and the anti-fogging function realizes a film temperature rise of 16.7 ${}^{\circ }$C in 5 min, reaching a maximum of 41.3 ${}^{\circ }$C. In this work, a fully automatic CIS film-driven algorithm based on PSNR is provided and the feasibility is verified by the driving dust removal module. The verification results within 48 h showed that the dust removal operation was successfully triggered under the artificially applied dust change and the PSNR cleaned to the initial value. For the algorithm, the PSNR can be used not only to measure the efficiency of dust removal and anti-fogging functions, but also as a quantitative parameter to determine when the dust removal function or anti-fogging function should be triggered. The whole working process does not need manual assistance, which could be widely used in outdoor optical device applications.

## Data Availability

The data presented in this study are available on request from the corresponding author.
